# Two Decapod Crustaceans, *Panopeus herbstii* and *Petrolisthes armatus*, Stabilize Their Gaze Using Achromatic Visual Cues, but Not the Angle of Linearly Polarized Light

**DOI:** 10.1093/iob/obaf034

**Published:** 2025-08-18

**Authors:** M Janakis, S Guirges, A C Grant, I Paris, E F LoPresti, D I Speiser

**Affiliations:** Department of Biological Sciences, University of South Carolina, 715 Sumter Street, Columbia, SC 29208, USA; Department of Biological Sciences, University of South Carolina, 715 Sumter Street, Columbia, SC 29208, USA; Department of Biological Sciences, University of South Carolina, 715 Sumter Street, Columbia, SC 29208, USA; Department of Biological Sciences, University of South Carolina, 715 Sumter Street, Columbia, SC 29208, USA; Department of Biological Sciences, University of South Carolina, 715 Sumter Street, Columbia, SC 29208, USA; Department of Biological Sciences, University of South Carolina, 715 Sumter Street, Columbia, SC 29208, USA

## Abstract

Gaze stabilization is important to animals because it allows them to visually differentiate between their own motion relative to their environment and the motion of objects within their environment. Animals can struggle to stabilize their gaze in environments that have a high amount of visual noise. In shallow aquatic environments, such as tidal creeks, the motion of the water's surface can create dynamic spatiotemporal fluctuations in illumination referred to as “caustic flicker.” This type of visual noise can create false-motion cues. To overcome this obstacle, shallow-dwelling aquatic animals may use color or polarized cues to stabilize their gaze rather than achromatic cues. Tidal creeks are often spectrally narrow due to light absorption by suspended particles such as algae, making color vision unreliable. Instead of using achromatic or chromatic cues, we hypothesize that crabs in tidal creeks stabilize their gaze using cues that vary in their angle of linear polarization (AoLP). To ask whether crabs from tidal creeks may use AoLP cues in gaze stabilization, we investigated polarization sensitivity in the Atlantic mud crab, *Panopeus herbstii*, and the green porcelain crab, *Petrolisthes armatus*. Using optomotor behavioral assays, we found that both porcelain and mud crabs use achromatic cues for gaze stabilization, but neither use AoLP cues across a range of light conditions that varied in spectral width, spectral peak, and total irradiance. Our findings are further evidence that although animals may theoretically benefit from using AoLP cues for gaze stabilization in visually noisy aquatic habitats, decapod crustaceans from tidal creeks seem to rely on achromatic cues for this task.

## Introduction

Tidal creeks are highly productive and ecologically vital environments that serve as nurseries for many species and as feeding grounds for many others (Holland et al. 2004; Sanger et al. 2015). Despite the ecological importance of tidal creeks, we know very little about the visual ecologies of the animals that live in them. Tidal creeks present a variety of challenges to vision, including high levels of visual noise and conditions that lead to low visual contrast. Ripples at the water's surface, for example, create visual noise underwater by refracting light, resulting in high-frequency spatiotemporal variation in illumination, a phenomenon known as caustic flicker (Lythgoe 1988; Collin et al. 2000; Venables et al. 2022). In addition to visual noise caused by caustic flicker, chromatic contrast in tidal creeks can be reduced when suspended particles selectively absorb certain wavelengths of light (Lythgoe 1988; Warrant and Johnsen 2013; Rose et al. 2019). Phytoplankton blooms, for instance, may add a spectrally narrow green filter to a scene, making it difficult for animals with color vision to distinguish between similar hues. Suspended particles can also scatter light, creating a veil of brightness which reduces contrast across visual scenes (Lythgoe 1988; Warrant and Johnsen 2013).

An important visual task for many animals, including those in tidal creeks, is gaze stabilization. To reliably detect moving objects, such as predators or prey, or static visual features, such as burrows, animals must differentiate between their own motion relative to their environment (egocentric motion) and the motion of objects within their environment (allocentric motion) (Nalbach 1990; Land and Nilsson 2012). To distinguish between egocentric and allocentric motion, animals stabilize their gaze by orienting to static visual landmarks (Srinivasan et al. 1999; Drerup and How 2021). Orienting to static landmarks can be difficult in tidal creeks because of caustic flicker and conditions that cause low visual contrast. Caustic flicker is a challenge for gaze stabilization because animals may mistake the movement of light for the motion of a nearby static object (Matchette et al. 2018; Drerup et al. 2024). Viewing conditions that decrease visual contrast are a further challenge to gaze stabilization because they make objects harder to distinguish at a distance, giving animals fewer options for reliable visual landmarks.

How may animals in tidal creeks reliably stabilize their gaze despite the challenges posed by caustic flicker and low visual contrast? Caustic flicker modulates light intensity, making achromatic visual cues unreliable and narrow spectral conditions reduce the reliability of chromatic visual cues. Thus, compared to achromatic or chromatic visual cues, polarized, cues may be a relatively reliable source of information for gaze stabilization in tidal creeks (Lythgoe 1988; Venables et al. 2022). Light is linearly polarized when the majority of its waves oscillate along the same axis. Aspects of linearly polarized light to which animals can be sensitive include the angle of linear polarization (AoLP) and the degree of linear polarization (DoLP) (Land and Nilsson 2012; Cronin et al. 2014). Animals from other types of shallow aquatic habitats have been shown to use linearly polarized light cues to inform a range of visually influenced behaviors (Glantz 2008; Marshall and Cronin 2014; How et al. 2015; Patel et al. 2024; Smithers et al. 2024). Cephalopods and crustaceans, for example, use polarization vision to compensate for false motion cues produced by caustic flicker, an effective approach because while caustic flicker modulates the intensity of light, it does not affect its polarization (Venables et al. 2022; Drerup et al. 2023; Drerup et al. 2024).

Crabs inhabit a wide range of environments, each with distinct visual challenges. We were curious if crabs that live in tidal creeks use the same types of visual cues for gaze stabilization as those from other types of littoral habitats. Most species of crab, like many other decapod crustaceans, are sensitive to linearly polarized light (Marshall and Cronin 2014; Patel et al. 2024) and they use this sensitivity to inform tasks including target detection (How et al. 2015; Drerup and How 2021), navigation (Goddard and Forward 1991), and signaling to conspecifics (Chiou et al. 2011). Polarization sensitivity is likened to color vision in invertebrates (Bernard and Wehner 1977; Labhart 2016; Basnak et al. 2018) and previous studies have demonstrated that gaze stabilization in other animals tends to be “color blind” in that it relies on intensity-based cues rather than those based on color or polarization contrasts (Schaerer and Neumeyer 1996; Krauss and Neumeyer 2003; Yamaguchi et al. 2008). Although most decapod species tested so far do not use cues that contrast in AoLP or DoLP for gaze stabilization (Drerup and How 2021), some evidence suggests that certain crustaceans may be able to use AoLP to stabilize their gaze (Glantz 2008).

**Fig. 1:**
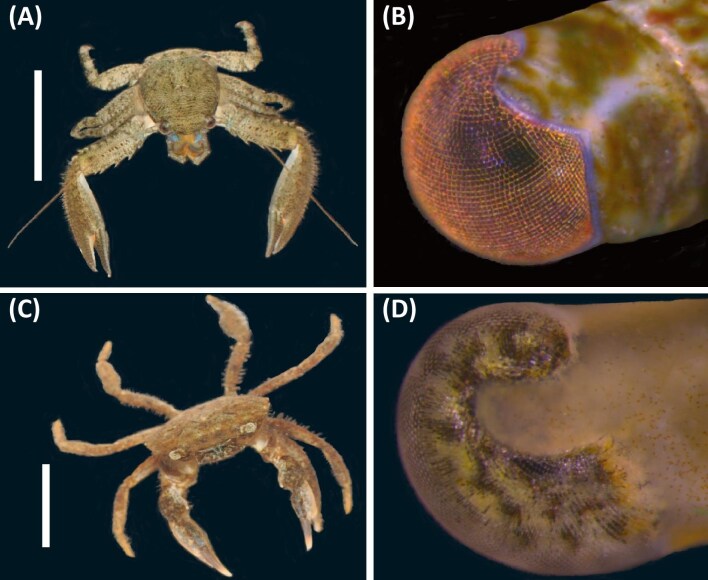
The green porcelain crab *Petrolisthes armatus* and the Atlantic mud crab *Panopeus herbstii.* (**A**) Dorsal view of *P. armatus.* (**B**) Ventral view of an eye from *P. armatus*. (**C**) Dorsal view of *P. herbstii*. (**D**) Ventral view of an eye from *P. herbstii*. The scale bars in (**A**) and (**C**) represent 1 cm.

To ask if crabs that dwell in tidal creeks stabilize their gaze using visual cues that vary in achromatic contrast or AoLP contrast, we tested the behavioral responses of the Atlantic mud crab, *Panopeus herbstii* (Fig. 1A and B), and the green porcelain crab, *Petrolisthes armatus* (Fig. 1C and D), to a series of optomotor stimuli. To ask if crabs use achromatic cues for gaze stabilization and to estimate visual acuity, we presented achromatic stimuli that varied in angular width. We predicted both crab species have relatively coarse spatial resolution (∼5°), similar to other shallow-dwelling decapod crustaceans (Baldwin and Johnsen 2011; Caves et al. 2016; Kingston et al. 2019). Using optomotor behavioral assays (Baldwin and Johnsen 2011; Caves et al. 2016; Kingston et al. 2019) with polarized stimuli (Darmaillacq and Shashar 2008; Bernáth and Meyer-Rochow 2016; Drerup and How 2021), we tested if crabs use cues that vary in AoLP for gaze stabilization. We anticipated that mud crabs would stabilize their gaze using AoLP cues because *P. herbstii* larvae have been shown to orient their body axes to the angle of polarization of linearly polarized light (Bardolph and Stavn 1978). We anticipated that porcelain crabs would not respond to cues that vary in AoLP because previous work suggests that their eyes have rhabdoms with microvillar arrangements that decrease polarization sensitivity (Eguchi et al. 1982). We also tested if mud crabs use AoLP cues for gaze stabilization under dim narrow-spectrum light or bright narrow-spectrum light, as these conditions are ecologically relevant for animals that live in aquatic habitats prone to phytoplankton blooms and other fluctuations in suspended materials. By conducting these experiments, we sought to learn how animals stabilize their gaze in tidal creeks, ecologically vibrant environments that present particularly challenging visual conditions to their inhabitants.

## Methods

### Animal collection and care

We collected green porcelain crabs, *Petrolisthes armatus* (Gibbes, 1850), and Atlantic mud crabs, *Panopeus herbstii* (H. Milne-Edwards, 1834), from Oyster Landing (33°20’58.5″N, 79°11′19.2″W) in North Inlet, South Carolina between January 2023 and January 2025. We collected crabs using hand nets during daytime low tides from water less than 1 m deep. We then transported animals to the University of South Carolina (Columbia, SC) in natural seawater (NSW) with aeration. Upon arrival, we housed animals individually in NSW at room temperature (22°C) and a salinity of 35 ppt with artificial burrows provided for shelter. We changed water and fed animals every 2 weeks. For food, porcelain crabs received a few drops of Marine Snow (Two Little Fishies, Miami Gardens, FL) and mud crabs received a few pellets of Hikari Crab Cuisine (Kyorin Food Ind. Ltd, Himeji, Japan). The project extended over several years, but each experiment involved crabs that had been in captivity for less than 2 months. Each experiment lasted 2 weeks or less.

### Equipment and procedures for optomotor experiments

We estimated visual acuity and tested gaze stabilization responses in crabs using optomotor behavioral assays, a well-established method for testing the visual abilities of invertebrates (McCann et al. 1997), including decapod crustaceans (Baldwin and Johnsen 2011; Caves et al. 2016; Kingston et al. 2019; Drerup and How 2021). These behavioral assays track the responses of animals to rotating vertical stripes. In our experiments, we mounted visual stimuli to the outside of a clear acrylic cylinder with a 30 cm diameter that we rotated around a smaller stationary cylinder (25 cm diameter) containing a third cylinder (12.5 cm diameter) containing a crab. Using polarized filters we verified that none of the acrylic cylinders were birefringent. We filled both of the stationary cylinders with NSW to ensure the angular widths of the stimuli, as viewed by a crab, remained as we intended. We rotated the 30 cm cylinder around the stationary cylinders using a bipolar high torque stepper motor (OMC Corporation Limited, Nanjing City, China) controlled by an Arduino Uno microcontroller with an attached motor shield (Adafruit, New York, NY). We programmed the Arduino to rotate the outermost cylinder 3 rotations/min for 40 s clockwise before reversing and rotating another 40 s counterclockwise, a speed we found was effective in preliminary trials.

All trials were lit from above with LEDs whose light we diffused with two pieces of filter paper (3000 Tough Rolux and 3027 Half Tough White Diffusion; Rosco Laboratories, Stamford, CT). We measured the absolute irradiance of light conditions (integrated from 400 to 700 nm) in our experiments using a spectrometer system with components from Ocean Optics (Dunedin, FL), including a Flame-S-VIS-NIR-ES spectrometer, a QP400-1-UV-VIS optical fiber, and a CC-3 cosine corrector. The absolute spectral response of the spectrometer was calibrated using an HL-3P-CAL Vis-NIR calibrated light source and the spectrometer was operated using OceanView software. In all four of our experiments, each animal experienced every optomotor stimulus once (unless they died prematurely). We presented one optomotor stimulus per day in a randomized order to each animal. We recorded trials from above using a GoPro Hero 6 (GoPro Inc., San Mateo, CA).

### Experiment 1: Responses of mud and porcelain crabs to achromatic and polarized optomotor stimuli under broad-spectrum light

In our first experiment, we tested visual acuity in mud crabs (*N* = 24) and porcelain crabs (*N* = 42) and asked if these animals stabilize their gaze using achromatic cues, AoLP cues, or both. To do so, we exposed crabs to six different optomotor stimuli: achromatic (black and white) stripes with angular widths of 2, 5, or 10° (as viewed from the center of the behavioral arena), an achromatic control stimulus of a uniform 50% gray, an experimental polarized stimulus, or a control polarized stimulus. We built the experimental and control polarized stimuli using a linearly polarized filter (43% transmission, American Polarizers, Inc., AP42–007T) and following established methods for testing responses to AoLP in other invertebrates (Darmaillacq and Shashar 2008; Bernáth and Meyer-Rochow 2016). For the experimental polarized stimulus, we created a horizontal-vertical polarization pattern using strips cut from the same polarized filter oriented so their AoLP alternated between 0° and 90° with respect to the vertical. For the control polarized stimulus, we arranged all strips so their AoLP was 0° with respect to the vertical. All of these strips of polarized filter had angular widths of 10° as viewed from the center of the behavioral arena. We backed both the experimental and control polarized stimuli with aluminum foil (Bernáth and Meyer-Rochow 2016). To check for visual artifacts, we compared the appearances of the experimental and control polarized stimuli from the crab's position in the behavioral arena using a GoPro camera. We took a first video with a linearly polarized filter placed in front of the camera lens, and then a second video without the filter (Supplementary  Video 1). We lit trials from above with an Aqua Illumination Prime HD LED fixture (C2 Development Inc., Ames, IA, USA; output 400–700 nm) adjusted with myAI (C2 development, Inc., Ames, IA) to provide an absolute spectral irradiance of downwelling light at the crab's position of 2.16^14^ photons cm^−^^2^ s^−^^1^ (∼165 lux), a light level that crabs likely experience naturally in tidal creeks (Supplementary Fig. 1A).

### Experiment 2: Responses of mud crabs to AoLP cues under dim narrow-spectrum light

In our second experiment, we asked if mud crabs stabilize their gaze using AoLP cues under relatively dim narrow-spectrum light. To do so, we tested the optomotor responses of naive mud crabs (*N* = 24) to the experimental and control polarized stimuli under four different colors of relatively dim narrow-spectrum light, for a total of eight treatments per individual crab. We produced these colors, which had peak irradiance values of near-UV (410 nm), blue (450 nm), green (515 nm), and red (660 nm), with the same light fixture described above. Using myAI, we adjusted output from the different sets of LEDs in the light fixture so that all produced an absolute spectral irradiance of downwelling light at the crab's position in the behavioral arena within 2% of our target irradiance of 1.52 × 10^13^ photons cm^−^^2^ s^−^^1^ (Supplementary Fig. 1B).

### Experiment 3: Responses of mud crabs to AoLP cues under bright narrow-spectrum light

In our third experiment, we asked if mud crabs stabilize their gaze using AoLP cues under relatively bright narrow-spectrum light. To do so, we tested the optomotor responses of naive mud crabs (*N* = 24) to the experimental and control polarized stimuli under the same four colors of narrow-spectrum light as in our second experiment, but under brighter conditions. We lit these trials from above using an Aqua Illumination Hydra 32HD LED fixture (C2 Development, Inc., Ames IA; output 400–700 nm). Again using myAI, we adjusted output from the different sets of LEDs in the light fixture so that all produced an absolute spectral irradiance of downwelling light at the crab's position in the behavioral arena within 1% of our target irradiance of 5.21 × 10^14^ photons cm^−^^2^ s^−^^1^ (Supplementary Fig. 1C).

### Experiment 4: Responses of mud crabs to AoLP cues under bright narrow-spectrum light and without overhead cues

In our fourth and final experiment, we asked if mud crabs in our earlier experiments may have perceived differences in intensity between the horizontally and vertically oriented strips of polarized filter in the experimental polarized stimulus. These differences in intensity are not apparent when the stimulus is viewed at a 0° angle (with respect to the horizontal), but when the polarized filters are viewed from an angle substantially away from 0°, their different axes of transmission may allow perceptibly different amounts of light through (Lenth 2024) (Supplementary  Video 1, panel 3). Thus, the mud crabs in our earlier experiments may have appeared to have been responding to AoLP cues when they were instead responding to intensity-based cues. To test this possibility, we recorded the optomotor responses of naive mud crabs (*N* = 20) to the experimental and control polarized stimuli under blue light (peak emission at 450 nm) with an irradiance at the crab's position in the behavioral arena of 5.21 × 10^14^ photons cm^−^^2^s^−^^1^. The critical difference between Experiment 4 and the preceding experiments was raising the positions of crabs within the optomotor apparatus so that they could only see the top 15 cm of the polarized optomotor stimuli and were thus unable to view the alternating strips of polarized filter at a steep angle (as they could in our first, second, and third experiments).

### Optomotor data analysis

We scored behavioral trials by recording the amounts of time animals moved in the same direction and at the same angular velocity as the rotating visual stimulus (following), moved in the opposite direction of the stimulus (anti-following), or stayed stationary. We only measured optomotor responses here (and not optokinetic responses) because *P. armatus* do not have mobile eyestalks and we have never observed *P. herbstii* following visual stimuli with their eyes. Trials were scored manually and all trials from each experiment were scored by the same individual. We computed a metric for optomotor responses that penalized against anti-following of the form: following index = (time following—time anti-following)/(time following + time anti-following + time stationary). Next, we used the lme4 package (Bates et al. 2015) in R version 4.4.0 (2024s-04-24) to generate linear mixed effects (LME) models that tested how the following index (the response variable) was affected by the type of stimulus presented (as a categorical predictor). In all models, individual animals were included as a random effect. Even though our data are bounded, we chose to use linear regressions because our data do not fit any standardized generalized linear distributions of residuals (binomial, Poisson, logistic, etc.). Additionally, there were few values at either −1 or 1 (the bounds of the variable for “following index”) and the residuals adequately fit the assumptions of the linear models by having normal distributions.

For the first experiment, which tested the responses of crabs to achromatic and polarized stimuli under bright broad-spectrum light, we created separate models for each species, both defined as (following index ∼ stimuli + 1|crab individual). We set the following index of the achromatic control stimulus as our reference group for each model. If the 95% confidence interval of the model estimate for a stimulus did not overlap with zero, we considered the following index of that stimulus to be significantly different from the following index for the achromatic control. We then performed Tukey's tests to compare the estimated marginal means of the following index of the control achromatic stimulus to the following indices of the 2°, 5°, and 10° achromatic stimuli using the “emmeans” package (Lenth 2024). We used this same package to then compare the following index of the control polarized stimulus to the following index of the experimental polarized stimulus.

In our second and third experiments, we tested the responses of mud crabs to the experimental and control polarized stimuli under narrow-spectrum light. In the second experiment the light was relatively dim, while in the third experiment it was ∼30 times brighter. For each of these experiments we created a LME model that predicted the following index of a stimulus depending on the color of light (near-UV, blue, green, or red) and polarized stimulus type (experimental or control): (following index ∼ stimuli*color + 1|crab individual). To ask if the responses of crabs to experimental and control polarized stimuli differed significantly under particular colors of light, we performed Tukey's tests (Lenth 2024).

In our fourth and final experiment, we only had two conditions (experimental and control polarized stimuli under blue light), so we compared the following index for each stimulus using a Welch two-sample *t*-test.

## Results

### Experiment 1a: Porcelain crabs and mud crabs have visual spatial resolutions between 4 and 10°

**Fig. 2:**
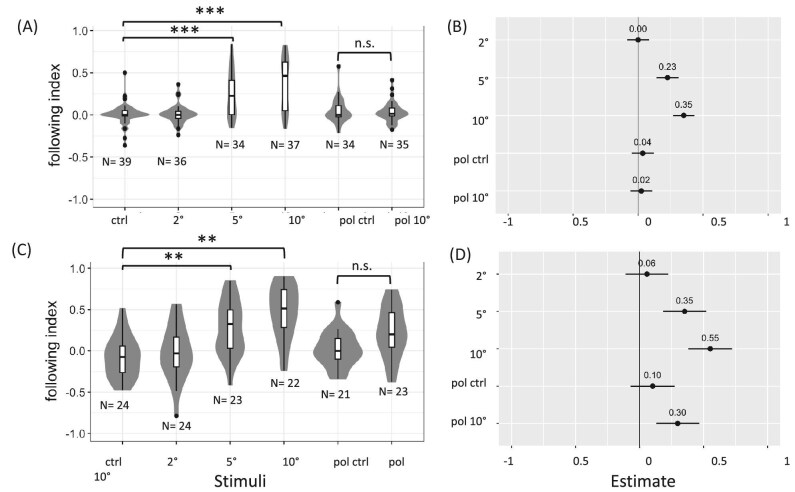
Results from behavioral trials with achromatic and polarized optomotor stimuli for porcelain crabs (**A** and **B**) and mud crabs (**C** and **D**). (**A**) Box plots show medians plus the interquartile range (IQR), whiskers are the lowest and highest values within 1.5 times the IQR from the upper and lower quartiles, and outliers are shown as dots. The violin plots show the distributions of following index for porcelain crabs viewing the achromatic (control, 2°, 5°, and 10°) and polarized (control and experimental) stimuli with sample sizes noted. (**B**) Linear mixed effect model estimates for porcelain crabs displaying 95% confidence intervals compared to responses to the achromatic control (represented by the vertical line). Values show the following index for a stimulus compared to the following index for the achromatic control stimulus, with a positive value denoting an increase in following index. ^**^ indicates *P* ≤ 0.005 and ^***^ indicates *P* ≤ 0.001 for Tukey's tests. (**C** and **D**) Corresponding figures for mud crabs.

In our first experiment, we found that both porcelain crabs and mud crabs have spatial vision with an angular resolution between 4 and 10°. According to estimates from our model, the following index of porcelain crabs (Fig. 2A and B) for achromatic visual stimuli with stripes of angular widths of 5° (*P* ≤ 0.001, df = 178, *t*-stat= −5.28) or 10° (*P* ≤ 0.001, df = 176, *t*-stat= −8.37) was significantly greater than the following index of the achromatic control stimulus. Similarly, the following index of mud crabs (Fig. 2C and D) for 5° (*P* ≤ 0.005, df = 108, *t*-stat= −4.14) and 10° (*P* ≤ 0.001, df = 108, *t*-stat= −6.42) achromatic stimuli was significantly greater than the following index of the achromatic control stimulus. The following index for the 2° achromatic stimulus was not significantly different from the following index of the achromatic control stimulus for either species (porcelain crabs: *P* = 1.0, df = 174, *t*-stat = −0.01; mud crabs: *P* = 0.99, df = 106, *t*-stat = −0.69). As measures of achromatic spatial resolution, we estimated the minimum resolvable angles in porcelain and mud crabs to be greater than 4° (twice the width of the widest stripes they did not follow) and less than or equal to 10° (twice the width of the narrowest stripes they did follow).

### Experiment 1b: Neither mud crabs nor porcelain crabs respond to AoLP cues under broad-spectrum light

From our first experiment, we also found that neither porcelain crabs nor mud crabs responded to the experimental polarized stimulus differently than they responded to the control polarized stimulus. Porcelain crabs did not have a higher following index when they viewed the experimental polarized stimulus than when they viewed the polarized control stimulus (*P* = 0.99, df = 178, *t*-stat = 0.24). Mud crabs had a higher following index when they viewed the experimental polarized stimulus than when they viewed the control polarized stimulus, but this difference was not significant (*P* = 0.23, df = 108, *t*-stat = −2.22).

### Experiments 2 and 3: Mud crabs appear to respond to cues that vary in AoLP under bright narrow-spectrum near-UV and blue light

**Fig. 3:**
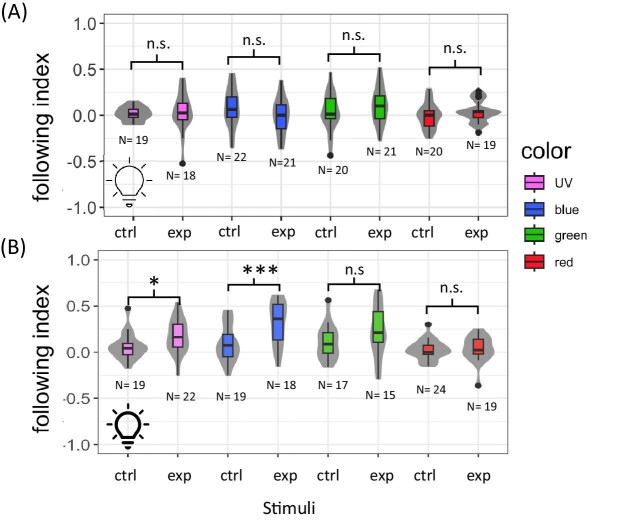
Responses of mud crabs to polarized optomotor stimuli under spectrally narrow conditions. The violin plots show the distributions of following index for mud crabs viewing the experimental (exp) and control (ctrl) polarized optomotor stimuli under different spectrally narrow light conditions (near-UV, blue, green, and red). The sample size is displayed for each treatment (values vary because some crabs died prematurely). The box plots show medians plus the interquartile range (IQR), whiskers are the lowest and highest values that are within 1.5 times the IQR from the upper and lower quartiles, and outliers are shown as dots. For each color condition we compared the following index of crabs viewing the experimental polarized stimulus to the following index of crabs viewing the control polarized stimulus using a pairwise Tukey's test (n.s. indicates *P* > 0.05, ^*^ indicates *P* < 0.01, ^***^ indicates *P* < 0.0001). (**A**) Results for trials conducted under dimmer narrow-spectrum conditions (1.52 10^13^ photons cm^−^^2^ s^−^^1^). (**B**) Results for trials conducted under brighter narrow-spectrum conditions (5.21 10^14^ photons cm^−^^2^ s^−^^1^).

Mud crabs had a higher following index in response to the experimental polarized stimulus than they did in response to the control polarized stimulus under narrow-spectrum near-UV or blue light, but only when the light was bright enough. In our second experiment (Fig. 3A), conducted under relatively dim conditions, the following index for crabs viewing the experimental polarized stimulus was not significantly greater than it was for crabs viewing the control polarized stimulus under any of the four color conditions we tested (near-UV: *P* = 0.64, df = 161, *t*-stat = 0.47; blue: *P* = 0.08, df = 161, *t*-stat = −1.74; green: *P* = 0.17, df = 161, *t*-stat = 1.38; red: *P* = 0.44, df = 161, *t*-stat = 0.77).

In our third experiment, (Fig. 3B), conducted under relatively bright conditions, the following index for mud crabs viewing the experimental polarized stimulus was significantly greater than the following index for animals viewing the control polarized stimulus under near-UV light (*P* = 0.03, df = 125, *t*-stat = 2.20) or blue light (*P* < 0.0001, df = 126, *t*-stat = 4.11). Under green light (*P* = 0.05, df = 130, *t*-stat = 1.95) or red light (*P* = 0.80, df = 127, *t*-stat = 0.25), the following index of the experimental polarized stimulus was not significantly different than the following index of the control polarized stimulus.

### Experiment 4: Mud crabs do not respond to AoLP cues when their viewing angle is limited

In experiment four, we limited the angles at which crabs could view the polarized stimuli. Mud crabs had a following index of 0.09 when viewing the control stimulus and a following index of 0.13 when viewing the experimental polarized stimulus. A Welch's two-sample *t*-test showed that these responses are not significantly different (*P* = 0.54, df = 37.99, *t*-stat = −0.60).

## Discussion

### Both crab species use achromatic cues for gaze stabilization and have coarse visual spatial resolution

In our first experiment, we found that mud crabs and porcelain crabs both exhibit optomotor responses to achromatic visual stimuli that are consistent with gaze stabilization. Additionally, we found these species have similar achromatic visual spatial resolutions. Both species of crab demonstrated spatial resolutions that fall between 4 and 10°, which is similar to spatial acuity observed in other benthic crustaceans of similar size, such as snapping shrimp and cleaner shrimp (Caves et al. 2016; Kingston et al. 2019). We acknowledge these are rough estimates of visual acuity and we hope future studies seek more precise measurements. We expect that coarse spatial resolution vision combined with living in turbid conditions results in crabs only being able to visually detect objects at a relatively close range. It is likely that these crustaceans use other modalities for longer-range sensing and use spatial vision at closer distances for tasks such as mate selection, predator detection, and shelter-seeking (Hines 2003; Dalesman and Inchley 2008)*.*

### Crabs do not use AoLP cues for gaze stabilization

We anticipated that porcelain crabs would not stabilize their gaze using linearly polarized cues while mud crabs would. The eyes of crustaceans are often sensitive to AoLP because of the structure of their rhabdoms, which are composed of photosensitive elements called microvilli, small finger-like projections of retinular cells (Cronin et al. 2014). In the apposition or reflecting superposition compound eyes found in most crustaceans, the rhabdoms are formed from bundles of microvilli that are inherently sensitive to waves of light oscillating parallel to their long axes. This means that many crustaceans’ photoreceptors are intrinsically sensitive to polarization contrasts (Marshall and Cronin 2014). Unlike most other crustaceans, *P. armatus* has rhabdoms with relatively disorganized microvilli, which has led previous authors to predict that this species has limited sensitivity to the polarization of light (Eguchi et al. 1982), perhaps as a way to increase the light-gathering ability of its eyes (Marshall and Cronin 2014).

We ran a series of experiments testing if mud crabs use AoLP cues for gaze stabilization as a way to overcome the challenges to vision they face in tidal creeks. These challenges include caustic flicker, which reduces the reliability of achromatic cues, and narrow-spectrum light conditions, which reduce the contrast of chromatic cues (Warrant and Johnsen 2013; Venables et al. 2022). Compared to achromatic or chromatic cues, AoLP cues may be a relatively reliable source of visual information for crabs living in tidal creeks. Our first experiment showed that the following index of mud crabs for the experimental polarized stimulus was significantly different than their following index for the achromatic control stimulus, but not their following index for the control polarized stimulus (Fig. 2C, D). These results hinted that mud crabs may use AoLP cues for gaze stabilization, so in our second and third experiments we tested the responses of mud crabs to AoLP cues under narrow-spectrum light. The following index of mud crabs for the experimental polarized stimulus was not greater than their following index for the control polarized stimulus under any of the dim narrow-spectrum conditions we tested. In contrast, under blue and near-UV wavelengths when the light was relatively bright, the following index of mud crabs for the experimental polarized stimulus was greater than their following index for the control polarized stimulus.

**Fig. 4:**
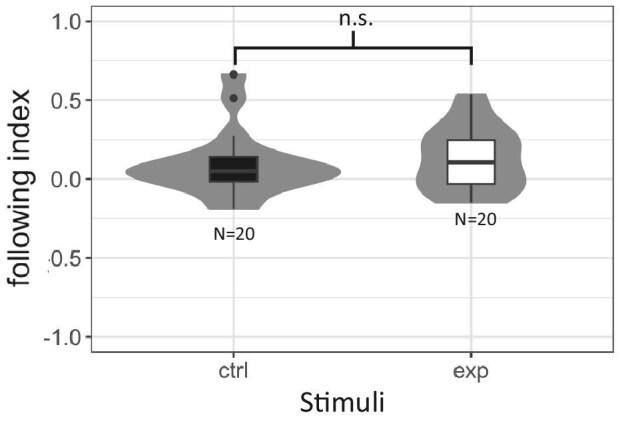
Responses of mud crabs to polarized optomotor stimuli when they could not view these stimuli at a steep angle. The violin plots show the distributions of the following index for mud crabs viewing the control (ctrl) and experimental (exp) polarized optomotor stimuli under bright narrow-spectrum blue light (5.21 10^14^ photons cm^−^^2^ s^−^^1^). Box plots show medians plus the interquartile range (IQR), whiskers are the lowest and highest values that are within 1.5 times the IQR from the upper and lower quartiles, and outliers are shown as dots. Sample size is denoted beneath each treatment. We compared the following index between stimuli using Welch's *t*-test (n.s. indicates *P* > 0.05).

We were intrigued to see evidence for gaze stabilization via AoLP cues in mud crabs, but we were wary that the responses of animals could have been influenced by their perception of unintended intensity-based cues. Experiments concerning the responses of animals to polarized light can be plagued by light artifacts that go unnoticed by the human eye (Foster et al. 2018). We conducted our fourth experiment because when orthogonally arranged polarized filters are viewed from an angle substantially away from 0° (with respect to the horizontal plane), their different axes of transmission may allow perceptibly different amounts of light through. Consequently, crabs viewing our experimental polarized stimulus at a steep angle could have perceived differences in intensity between adjacent strips of polarized filter. In our first three experiments, the maximum angle at which a crab could view the optomotor stimuli was ∼71°. In experiment four, we decreased the maximum viewing angle to less than 60°. When crabs were no longer able to view the polarized stimuli from as steep of an angle, we no longer observed mud crabs responding differently to the experimental and control polarized stimuli (Fig. 4). This suggests that mud crabs were not responding to AoLP contrast in our first three experiments, but were instead responding to low-contrast intensity-based cues. Mud crabs may have responded to low-contrast intensity-based cues under blue and near-UV light because their eyes are more sensitive to blue light than to other parts of the visual spectrum. Previous microspectrometry recordings have shown that photoreceptors in the eyes of *P. herbstii* have a peak absorption at 493 nm, blue (Cronin and Forward 1988).

### Gaze stabilization in invertebrates

Past studies across invertebrate groups have shown that gaze stabilization tends to be “color blind” (i.e., does not use chromatic cues) (Schaerer and Neumeyer 1996; Krauss and Neumeyer 2003; Yamaguchi et al. 2008). More recent research has investigated whether the same may be true for polarized cues. Current findings have shown that some invertebrate species stabilize their gaze with polarized cues while other closely related taxa do not. For example, the cuttlefish *Sepia elongata* (Darmaillacq and Shashar 2008) did not respond to optomotor cues that varied in AoLP, but its close relatives *Sepia plangon* and *Sepia mestus* did (Talbot and Marshall 2010). Among insects, the mosquito *Aedes aegypti* (Bernáth and Meyer-Rochow 2016) and the dragonfly *Anax imperators* (Sharkey et al. 2015) exhibit optomotor responses to polarized cues. In a study of AoLP responses among five species of decapod crustaceans, Drerup and How (2021) investigated two species of shrimp, *Palaemon elegans and P. serratus*, the hermit crab *Pagurus bernhardus*, the shore crab *Carcinus maenas*, and the fiddler crab *Afruca tangeri*. They found that none of these crustaceans stabilize their gaze using polarized light alone, but that all five species were able to use linearly polarized light cues to detect objects (Drerup and How 2021). In a separate set of experiments, the crayfish *Pacifasticus leniusculus* and *Procambrus clarkii* were found to respond physiologically and optokinetically to optomotor cues that varied in AoLP. These findings suggest that crayfish can detect AoLP cues, but it remains unclear whether they use them to stabilize their gaze (Glantz and Schroeter 2006; Glantz and Schroeter 2007; Glantz 2008). Our findings are generally consistent with those from prior studies: invertebrates can use AoLP for gaze stabilization and decapods are often sensitive to AoLP, but decapods do not appear to use AoLP cues for gaze stabilization. Future studies should examine whether mud crabs may use AoLP sensitivity for other purposes such as contrast enhancement for target detection in tidal creeks (How et al. 2015; Smithers et al. 2019; Venables et al. 2022). For example, sensitivity to AoLP may help mud crabs detect prey or predators under turbid conditions by dehazing images, a process which has been shown to increase sighting distances underwater (Cartron et al. 2013).

## Supplementary Material

obaf034_Supplemental_Files **Supplemental Fig. 1** Spectral irradiance of light conditions in behavioral experiments**.** (**A**) The first experiment, conducted under broad-spectrum light (2.1610^14^ photons cm^−^^2^ s^−^^1^); (**B**) The second experiment, conducted under relatively dim isoluminant narrow-spectrum light (1.52 10^13^ photons cm^−^^2^ s^−^^1^); and (**C**) the third experiment, conducted under relatively bright isoluminant narrow-spectrum light (5.21 10^14^ photons cm^−^^2^ s^−^^1^).
**Supplemental video**: **POV videos of polarized experimental and control stimuli.** The control (**A, B**) and experimental (**C, D**) polarized stimuli viewed from the position of the crab in the optomotor behavioral arena. (**A, C**) show stimuli viewed without polarized filter and (**B, D**) show the same stimuli viewed from the same location with a linearly polarized filter placed over the camera lens.

## Data Availability

The data underlying this article are available in the article and in its online supplementary material.
