# Supplementary figures and images for: Two Decapod Crustaceans, Panopeus herbstii and Petrolisthes armatus, Stabilize Their Gaze Using Achromatic Visual Cues, but Not the Angle of Linearly Polarized Light
Source: Integr Org Biol. 2025 Aug 18;7(1):obaf034. doi: 10.1093/iob/obaf034 (PMC12395132; doi:10.1093/iob/obaf034)

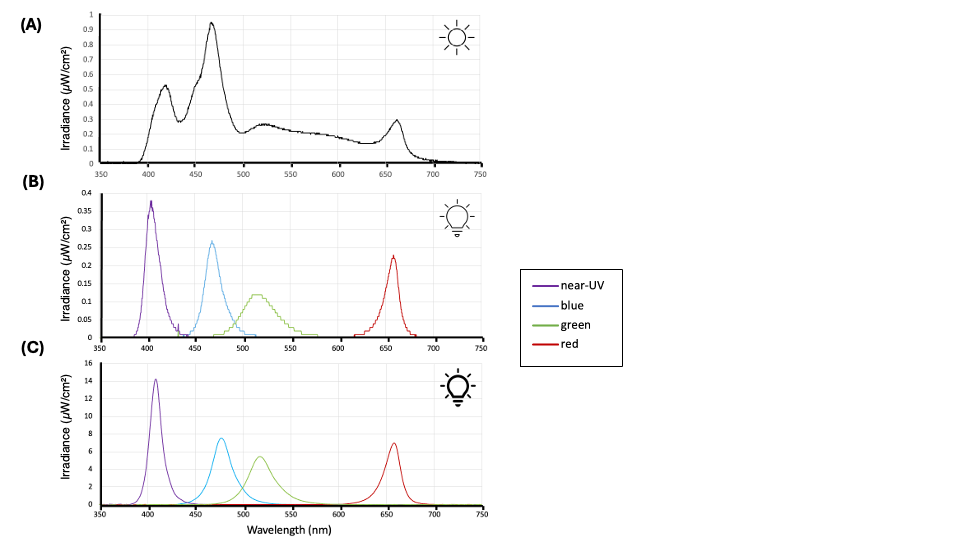

Supplement: obaf034_Supplemental_Files — Supplemental Fig. 1 Spectral irradiance of light conditions in behavioral experiments. (A) The first experiment, conducted under broad-spectrum light (2.161014 photons cm−2 s−1); (B) The second experiment, conducted under relatively dim isoluminant narrow-spectrum light (1.52 1013 photons cm−2 s−1); and (C) the third experiment, conducted under relatively bright isoluminant narrow-spectrum light (5.21 1014 photons cm−2 s−1). Supplemental video: POV videos of polarized experimental and control stimuli. The control (A, B) and experimental (C, D) polarized stimuli viewed from the position of the crab in the optomotor behavioral arena. (A, C) show stimuli viewed without polarized filter and (B, D) show the same stimuli viewed from the same location with a linearly polarized filter placed over the camera lens. [file obaf034_supplemental_files.zip › New_crab_opto_figs.tiff]
